# Impact of the COVID-19 Epidemic on a Pan-Asian Academic Oncology Clinical Trial

**DOI:** 10.1200/GO.20.00072

**Published:** 2020-04-15

**Authors:** Eva Segelov, Hans Prenen, Daphne Day, C. Raina Macintyre, Estelle Mei Jye Foo, Raghib Ali, Quanyi Wang, Xiaoting Wei, Gilberto de Lima Lopes, Kefeng Ding, Gong Chen, John Whay Kuang Chia, Han Chong Toh, on behalf of the ASCOLT Investigators

**Affiliations:** ^1^ Department of Oncology, Monash Health, and Faculty of Medicine, Monash University, Melbourne, Victoria, Australia; ^2^ Oncology Department, University Hospital Antwerp, Edegem, Belgium; ^3^ Kirby Institute, University of New South Wales, Sydney, New South Wales, Australia; ^4^ Department of Medical Oncology, National Cancer Centre Singapore, Singapore; ^5^ Public Health Research Centre, New York University, Abu Dhabi, United Arab Emirates; ^6^ Institute for Infectious Disease and Endemic Disease Control, Beijing Centre for Disease Prevention and Control, Beijing, People’s Republic of China; ^7^ Family Health International Clinical, Beijing, People’s Republic of China; ^8^ Global Oncology Program, Sylvester Comprehensive Cancer Center, University of Miami Miller School of Medicine, Miami, FL; ^9^ Department of Surgical Oncology, The Second Affiliated Hospital, Zhejiang University School of Medicine, Hangzhou, People’s Republic of China; ^10^ Department of Colorectal Surgery, Sun Yat-Sen University Cancer Centre, Guangzhou, People’s Republic of China

On January 30, 2020, the WHO declared a public health emergency of international concern for COVID-19, an epidemic novel strain of coronavirus that first infected humans in Wuhan, China.^1^ Over the following days and weeks, governments in China and across Asia started to implement drastic public health control measures, and Western countries began reporting patients as well.^2^ Measures have included quarantine, travel restrictions, social distancing (such as closing schools and banning mass gatherings), and personal hygiene measures, including wearing masks, implementing hospital infection control, and use of the International Health Regulations.^3^ To our knowledge, the impact of the epidemic on the running of clinical trials has not been reported to date. We describe the consequences of the COVID-19 epidemic, based on our experience with the ASCOLT trial (ClinicalTrials.gov identifier: NCT00565708), an academic phase III study open in 41 sites across 9 Asian countries and regions (mainland China, Hong Kong, India, Indonesia, Malaysia, Singapore, South Korea, Sri Lanka, and Taiwan).

The ASCOLT trial is evaluating the benefit of 3 years of aspirin versus placebo after standard adjuvant therapy for high-risk colorectal cancer. If positive, this could have a huge impact on reducing cancer deaths using an inexpensive and familiar medication. The trial was purposefully run within the Asia-Pacific region and specifically sought to engage low- and middle-income countries. At the start of 2020, 1,480 of a total of 1,587 patients had been recruited from 74 centers in 12 countries. A total of 447 patients were enrolled in mainland China, and 617 were from other Asian sites; the corresponding numbers of patients with COVID-19 were 78,497 and ranged from 0 to 1,766, respectively (Fig 1).^4^ There is concern that true patient numbers are higher because of under-reporting.^5^

**FIG 1:**
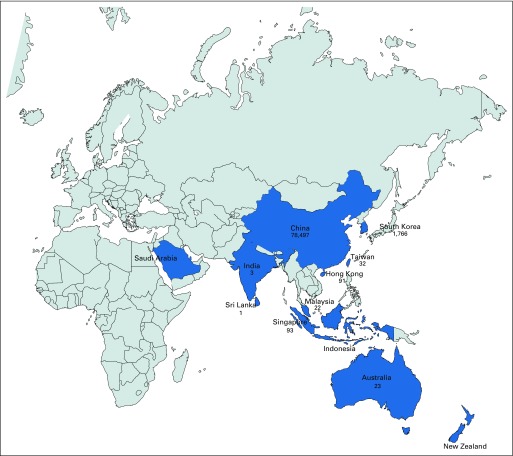
ASCOLT recruiting countries/regions and corresponding number of confirmed COVID-19 patients, as of February 28, 2020^4^. The number of COVID-19 cases for Hong Kong and Taiwan are listed separately.

The rapid onset and escalation of the coronavirus epidemic triggered health systems and governments to respond quickly, with public health concerns overriding standard practices. However, each jurisdiction enacted different restrictions, making it challenging for trial oversight. First, it has been difficult to systematically gather information regarding restrictions at each site and how they affect trial conduct. Second, there are no clear guidelines for Trial Management Committees on their ethical obligations under these circumstances, nor is there any guidance for investigators or trial participants. Third, despite public safety being paramount, the impact on clinical trials across all fields of medicine is potentially enormous, so that rational measures to prevent “throwing the baby out with the bathwater” are vital.

Clinical trials will be affected in multiple ways, and there may well be a different impact and response for academic studies (usually on shoestring budgets) compared with large, well-resourced industry-sponsored trials; interestingly, there has been little formal information regarding the latter. Recruitment may be reduced or halted, perhaps for a prolonged period. This will extend trial timelines and deplete already limited budgets, as running costs persist even during inactivity. Depending on stage, the viability of the trial may be threatened at that site, in that country, or even in its entirety. Logistic consequences include disruption of drug supply and increased loss to follow-up because of lockdown, travel restrictions, or patient preference to avoid hospitals. Statistical considerations include the impact of treatment disruptions on endpoints, including potential alteration of event rate, including death from all causes, higher rates of missing data, and delay in routine surveillance of endpoints, which could falsely increase disease-free survival rates. Human resources are affected by staff illness and quarantine, diversion of duties, cancellation or delay of research ethics and governance processes, restriction of monitoring visits, and cancellation of investigator meetings. Biospecimen collection protocols may be affected; for this particular outbreak, throat swab and saliva collections are being discouraged. Transport of tissues across international borders could be subject to even further stringencies.

The impact of the coronavirus outbreak on the ASCOLT trial has been significant. The 10 Chinese sites are located in Guangdong, Beijing, Shandong, Zhejiang, and Jiangsu (none in Hubei, the province where Wuhan is situated). Since January 23, all sites have suspended recruitment. From Guangzhou, one of the cities with the most patients with the virus outside of Hubei Province, G.C. reports, “All clinical trials at my center have been halted since February due to the virus outbreak. A total of 53 patients have been successfully recruited into the ASCOLT study, and 19 of them are still taking the study drug. All patients’ hospital visits are also halted; instead [we are using] phone-call follow-up and fax-back of investigation reports….6 patients need to get a new package of the study drug in February; we decided to deliver the package by post express....a new ICF [informed consent form] has been approved and signed by all patients.”

In Hong Kong, the study center pharmacy stopped dispensing oral research treatments, and trial visits are postponed. In Taiwan, there are no restrictions on visits, but personal hygiene measures and temperature checks for staff and patients are enforced. In Singapore, screening and recruitment for all clinical trials was halted by the National Cancer Centre Singapore hospital administration on February 10. Outpatients receiving treatment can continue scheduled visits; however, investigators are reporting a high rate of nonattendance; nonurgent appointments are discouraged, and it was recommended that they be deferred. Daily updates are issued from the regional health cluster SingHealth and Ministry of Health.

Many ASCOLT investigators in affected countries have reported on the emotional strain of COVID-19 in their workplace, including sick and overworked team members. One investigator sent a poem about volunteers: “I send my youngest ‘baby’ to the battlefield.” This is likely to affect trials because much of the work involved is in addition to usual duties.

Adaptations to facilitate the ongoing running of the ASCOLT trial have been pragmatic, based on ad hoc consultation initiated by sites with the chief ASCOLT Trial Manager. Practical changes, such as a switch to phone or local practitioner follow-up and mailing of medication kits, have so far not been submitted to a central process of protocol amendment; there is concern about imposing additional burden on strained health systems. A Trial Management Committee meeting is planned to endorse and standardize advice, as well as to consider the overall impact on the trial.

Clinical trials are fundamental for medical progress, but their vulnerability to external forces is highlighted by the recent coronavirus outbreak. Although management of the epidemic is the highest priority for affected hospitals, the impact on clinical trials, which themselves may lead to a major reduction in death rates from a vast spectrum of diseases, raises ethical issues with regard to the special duty of care owed to trial participants. The global clinical trial community has been caught without contingencies and should now start a formal process of “emergency planning” to endorse processes that can be applied to future unforeseen circumstances. A rational, carefully adapted policy encompassing operational responses to such crises would be of great utility. Beyond the crisis, reevaluating the dated model of repeated in-person visits at 1 site for trial participation should be encouraged, capitalizing on major advances in communication technology and big data. Finally, a reluctance to pursue trials, particularly academic research, in any particular region of the world for fear of epidemics would be perhaps one of the most disastrous outcomes of all.

## References

[1] WHO: Statement on the second meeting of the International Health Regulations (2005) Emergency Committee regarding the outbreak of novel coronavirus (2019-nCoV). https://www.who.int/news-room/detail/30-01-2020-statement-on-the-second-meeting-of-the-international-health-regulations-(2005)-emergency-committee-regarding-the-outbreak-of-novel-coronavirus-(2019-ncov)

[2] Munster VJ Koopmans M van Doremalen N et al A novel coronavirus emerging in China - key questions for impact assessment N Engl J Med 382 692 694 2020 31978293 10.1056/NEJMp2000929

[3] Heymann DL Shindo N: COVID-19: What is next for public health? Lancet 395 542 545 2020 32061313 10.1016/S0140-6736(20)30374-3 PMC7138015

[4] WHO: Coronavirus disease (COVID-2019) situation reports. Situation report - 38. https://www.who.int/docs/default-source/coronaviruse/situation-reports/20200227-sitrep-38-covid-19.pdf?sfvrsn=9f98940c_2

[5] Mallapaty S Scientists fear coronavirus spread in countries least able to contain it Nature 578 348 2020 32071443 10.1038/d41586-020-00405-w

